# Luteolin Prevents H_2_O_2_-Induced Apoptosis in H9C2 Cells through Modulating Akt-P53/Mdm2 Signaling Pathway

**DOI:** 10.1155/2016/5125836

**Published:** 2016-07-25

**Authors:** Hong Chang, Chun Li, Kuiyuan Huo, Qiyan Wang, Linghui Lu, Qian Zhang, Yong Wang, Wei Wang

**Affiliations:** ^1^Beijing University of Chinese Medicine, Bei San Huan Dong Lu 11, Chao Yang District, Beijing 100029, China; ^2^Traditional Chinese Medicine College, North China University of Science and Technology, No. 57 South Road, Tangshan, Hebei 063000, China; ^3^Modern Research Center for Traditional Chinese Medicine, Beijing University of Chinese Medicine, Bei San Huan Dong Lu 11, Chao Yang District, Beijing 100029, China

## Abstract

*Introduction.* Luteolin, a falconoid compound in many Chinese herbs and formula, plays important roles in cardiovascular diseases. The underlying mechanism of luteolin remains to be further elaborated.* Methods*. A model of hydrogen peroxide- (H_2_O_2_-) induced H9C2 cells apoptosis was established. Cell viabilities were examined with an MTT assay. 2′,7′-Dichlorofluorescin diacetate (DCFH-DA) and flow cytometry were used to detect ROS level and apoptosis rate, respectively. The expressions of signaling proteins related to apoptosis were analyzed by western blot and mRNA levels were detected by real-time polymerase chain reaction (PCR). Quercetin was applied as positive drug.* Results*. Incubation with various concentrations of H_2_O_2_ (0, 50, 100, and 200 *μ*M) for 1 h caused dose-dependent loss of cell viability and 100 *μ*M H_2_O_2_ reduced the cell viability to approximately 50%. Treatments with luteolin and quercetin protected cells from H_2_O_2_-induced cytotoxicity and reduced cellular ROS level and apoptosis rate. Moreover, luteolin could downregulate the expressions of Bax, caspase-8, cleaved-caspase-3, and p53 in apoptotic signaling pathway. Further study showed that the expressions of Akt, Bcl-2, and Mdm2 were upregulated by luteolin.* Conclusion*. Luteolin protects H9C2 cells from H_2_O_2_-induced apoptosis. The protective and antiapoptotic effects of luteolin could be mediated by regulating the Akt-P53/Mdm2 apoptotic pathway.

## 1. Introduction

Heart failure (HF) is the common condition resulting from a variety of cardiovascular diseases. The morbidity and mortality of HF remain high even though great efforts have been made to manage it [1]. The five-year survival rate of HF is only 35% [2]. One of the molecular mechanisms of HF is associated with apoptosis which occurs in various cardiovascular diseases, including myocardial infarction (MI), atherosclerosis (AS) and ischemia/reperfusion (IR) [3, 4]. Cardiomyocytes apoptosis is considered to be a key factor in the development of heart failure [5].

P53, known as the tumor suppressor gene, could induce apoptosis by blocking cellular DNA damage repair. Accumulating evidence shows that p53 plays a pivotal role in the progression of AS, MI, and IR [6, 7]. Expression of p53 is higher in the failing myocardium than normal cardiomyocytes [8]. By activating proapoptotic proteins, such as Bax and Bak, p53 induces apoptosis and accelerates progression of HF.

Murine double minute 2 (Mdm2), which is induced by p53, interacts with p53 and inhibits the apoptotic effect of p53. P53 level is regulated by the feedback between Mdm2 and p53 [9, 10]. A model of MI in mice [7] showed that a synthetic inhibitor targeting p53 could reduce cardiomyocytes apoptosis. Meanwhile, the area of MI was enlarged, indicating that the side effects of monotarget therapy should be taken seriously. Developing apoptotic inhibitors with minor side effects is an important potential strategy in the management of HF.

Traditional medicine has been making great contributions in treating varieties of human diseases including heart failure [11]. Luteolin, one of the flavonoids abundant in many vegetables and fruits [12], has been reported to provide protective effects against oxidative stress, inflammation, and cancer [13–15]. Plenty of evidence indicate that luteolin exerts multiple effects in cardiovascular diseases [16, 17]. In particular, luteolin has been shown to have antiapoptotic effects in cardiomyocytes [18, 19]. However, the antiapoptotic mechanism of luteolin is unclear and the effects of luteolin on Mdm2 and p53 remain to be investigated.

In this study, we explored the antiapoptotic effects of luteolin in H9C2 cell lines. Cellular apoptosis was induced by H_2_O_2_ culture and the effect of luteolin on p53/Mdm2 apoptotic signaling pathway was investigated.

## 2. Material and Methods

### 2.1. Material and Chemicals

MTT, DCFH-DA, and H_2_O_2_ (3 wt.%) were purchased from Sigma (USA). The Annexin V-PI kit was obtained from BD (USA). cDNA Synthesis kit, Green PCR Master Mix, penicillin, streptomycin, and 0.05% trypsin were purchased from Invitrogen (USA). Dulbecco's modified Eagle's medium (DMEM) was purchased from HyClone (USA). Fetal bovine serum (FBS) was purchased from Corning (USA). Luteolin and quercetin were purchased from National Institutes for Food and Drug Control (China). Luteolin and quercetin were dissolved in DMSO for in vitro assay as storage concentration (100 mM).

### 2.2. Cell Culture and Grouping

H9C2 cardiomyocyte cell line was obtained from China Infrastructure of Cell Line Resources (Beijing, China). The H9C2 cells were cultured in high glucose DMEM supplemented with 10% FBS and a mixture of penicillin (100 U/mL) and streptomycin (100 *μ*g/mL) in a humidified incubator with 5% CO_2_ at 37°C. Cells were nearly 80–90% confluent and treated with 5, 10, and 20 *μ*M luteolin and 10 *μ*M quercetin for 6 h. Then cells were divided into several groups: normal control group, H_2_O_2_-induced model group, luteolin-treated group, and quercetin-treated positive control group. Cells in model group were cultured in DMEM for 6 h, followed by treatment with H_2_O_2_ for 1 h. Cells in luteolin and quercetin groups were pretreated with luteolin and quercetin for 6 h before exposed to H_2_O_2_.

### 2.3. Establishment of Apoptosis Model Induced by H_2_O_2_

H9C2 cells were seeded in 96-well plates at a density of 10^4^ cells per well. Following overnight adherence, cells were incubated with H_2_O_2_ (0, 50 *μ*M, 100 *μ*M, and 200 *μ*M) in DMEM at 37°C for 1 h. Cells viabilities were determined by MTT assay. Cells were treated with MTT solution dissolved in DMEM (0.5 mg/mL) for 4 h. The supernatants were removed, followed by the addition of 150 *μ*L DMSO to each well to dissolve the precipitate. The absorbance of each well was then measured with a microplate reader (Thermo, USA) at a wavelength of 492 nm.

### 2.4. Cell Viability Analysis

H9C2 cells were seeded as described above. Before being treated with H_2_O_2_ for 1 h, cells were incubated with luteolin (5 *μ*M, 10 *μ*M, and 20 *μ*M) and quercetin (10 *μ*M) in DMEM supplemented at 37°C for 6 h. After treatment, MTT assay was taken to measure cell viability.

### 2.5. Cell Morphological Analysis

H9C2 cells were seeded at a density of 10^5^ cells/mL in 6-well plates. When the confluence reached 70–80%, cells were pretreated with/without luteolin (10 *μ*M) and quercetin (10 *μ*M) for 6 h and then exposed to H_2_O_2_ (100 *μ*M) for another 1 h. After incubation, cell morphology was photographed under an inversion microscope (OLYMPUS, Japan).

### 2.6. Analysis of Reactive Oxygen Species (ROS)

The cellular production of ROS was measured by detecting the fluorescent intensity of DCFH-DA, an oxidant-sensitive probe. After incubations at different conditions, H9C2 cells were washed three times with PBS, followed by incubation with DCFH-DA (10 *μ*M) at 37°C for half an hour. Fluorescent intensity was examined under a fluorescence microscope (OLYMPUS, Japan) with excitation at 485 nm and emission at 525 nm. Image J was used to analyze the data.

### 2.7. H9C2 Cell Apoptosis Assay

The Annexin V-PI method was used to detect the apoptosis rate of H9C2 cells by flow cytometry. Cells were harvested with 0.05% trypsin, washed three times with cold PBS (4°C), and collected by centrifugation at 1000 rpm for 5 minutes. After that cells were resuspended in 200 *μ*L binding buffer and incubated with Annexin V (10 *μ*g/mL) and PI (10 *μ*g/mL) in the dark for 15 minutes at room temperature. Then cells were detected with a flow cytometer (BD, USA).

### 2.8. Quantitative Real-Time PCR Analysis

The total mRNA of cells was extracted using TRIzol (Thermo, USA) and cDNA was synthesized from 2 *μ*g of total RNA using an RT kit (Thermo, USA) following the manufacturer's instructions. The mRNA quantity of Mdm2 gene was assessed by quantitative real-time PCR (ABI, USA). The primers used for amplification are as follows: *β*-actin F, CCCATCTATGAGGGTTACGC; *β*-actin R, TTTAATGTCACGCACGATTTC; Mdm2 F, TTGATGATGGCGTAAGTGA; Mdm2 R, AGGCTGTAATCTTCTGAGTC.

### 2.9. Western Blotting Analysis

The collected cells were prepared with RIPA buffer (PPLYGEN, China) and proteins were extracted according to the manufacture's instruction. Protein contents were measured with BCA Bradford protein assay (PPLYGEN, China). After addition of loading buffer, cell lysates were separated by 10% SDS-PAGE and transferred to NC membranes (Millipore, Germany). After being blocked with 5% nonfat dry milk for 2 h, the membrane was incubated with different primary antibodies (Abcam, USA) overnight at 4°C and washed with TBST three times. The membrane was then incubated with HRP-conjugated secondary antibodies (ZSGB-BIO, China) for 1 h at room temperature. After being washed with TBST for three times, the proteins were detected with an enhanced chemiluminescence agent (GE, USA) and quantified by densitometry using an image analyzer (Bio-Rad, USA). *β*-actin (ZSGB-BIO, China) served as an internal control.

The antibodies included rabbit monoclonal antibodies against cleaved-caspase-3 (1 : 1000), Bcl-2 (1 : 1000), Bax (1 : 1000), Akt (1 : 1000), rabbit multiclonal antibodies against caspase-8 (1 : 1000), mouse monoclonal antibody against p53 (1 : 1000), and secondary antibodies (goat anti-rabbit, 1 : 5000; goat anti-mouse, 1 : 5000).

### 2.10. Statistical Analyses

Data were expressed as the mean ± SD. Analysis was undertaken by one-way ANOVA. Differences between groups were considered as statistically significant when *P* < 0.05.

## 3. Result

### 3.1. Working Concentration of H_2_O_2_

**Figure 1:**
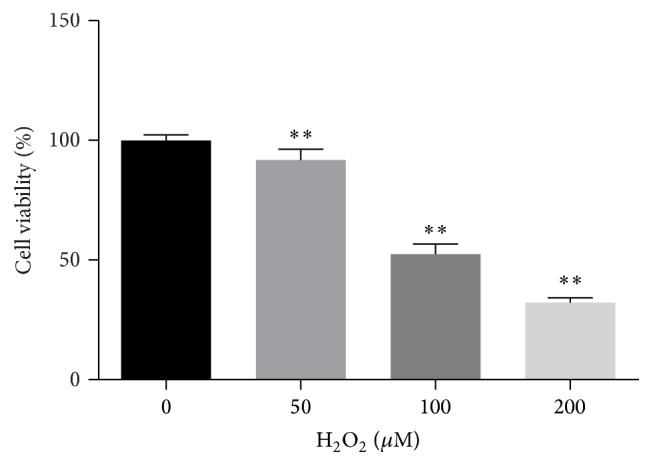
Cell viability in cells treated with different concentrations of H_2_O_2_ for 1 h. MTT assay was taken to measure cell viability. Data were expressed as mean ± SD. ^*∗∗*^ *P* < 0.01 versus control.

H9C2 cell lines were incubated with different concentrations of H_2_O_2_ for 1 h. Cell viability was measured by MTT assay. The result showed that H_2_O_2_ induced dose-dependent decrease of cell viability (Figure 1). When cells were incubated with 100 *μ*M and 200 *μ*M H_2_O_2_, cell viabilities were reduced significantly compared with control group (*P* < 0.01). The cellular survival rate in 100 *μ*M H_2_O_2_-treated group was 52.48 ± 0.0416% and these cells could be applied as apoptotic model to assess effects of luteolin. Therefore, 100 *μ*M H_2_O_2_ was applied to cells to induce apoptosis in the subsequent experiments.

### 3.2. Effects of Luteolin/Quercetin on Cell Survival Rate and H_2_O_2_-Induced Cytotoxicity

**Figure 2:**
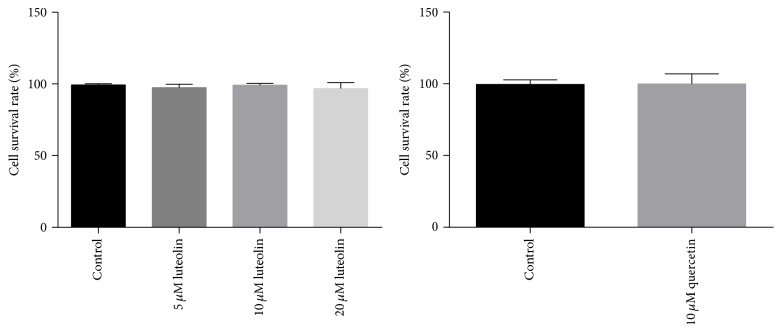
5, 10, and 20 *μ*M luteolin and 10 *μ*M quercetin had no effect on cell survival rate.

**Figure 3:**
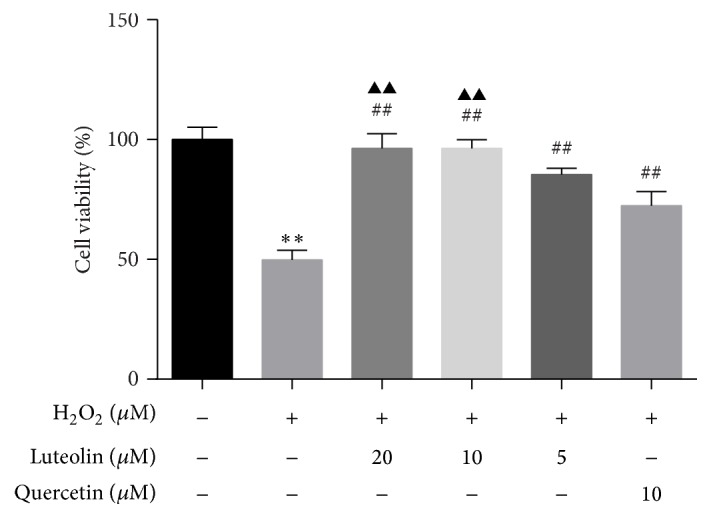
Luteolin rescued H_2_O_2_-induced loss of cell viability. H9C2 cells were preincubated with different concentrations of luteolin (5 *μ*M, 10 *μ*M, and 20 *μ*M)/quercetin (10 *μ*M) for 6 h and then treated with 100 *μ*M H_2_O_2_ for 1 h. Cell viability was detected with MTT assay. Data were expressed as mean ± SD. ^*∗∗*^ *P* < 0.01 versus control; ^##^ *P* < 0.01 versus model; ^▲▲^ *P* < 0.01 versus 5 *μ*M luteolin.

Cell viabilities were assessed by MTT assay. As shown in Figure 2, cell survival rates in luteolin (5, 10, and 20 *μ*M) and quercetin (10 *μ*M) groups were similar to that in control group, indicating that quercetin and luteolin have no cytotoxicity on H9C2 cells. The effects of luteolin/quercetin on H_2_O_2_-induced cytotoxicity were also detected. As shown in Figure 3, luteolin (5 *μ*M, 10 *μ*M, and 20 *μ*M)/quercetin (10 *μ*M) pretreatment provided significant protective effects on H_2_O_2_-mediated cytotoxicity in a dose-dependent manner (^##^ *P* < 0.01). Cells treated with 10 *μ*M luteolin had significant higher viability than those treated with 5 *μ*M luteolin and cell viabilities were similar between cells treated with 10 *μ*M and 20 *μ*M luteolin. Therefore, 10 *μ*M luteolin was applied to treat cells to assess its effects in the subsequent experiments.

### 3.3. Effects of Luteolin on H_2_O_2_-Induced Morphologic Changes in H9C2 Cells

**Figure 4:**
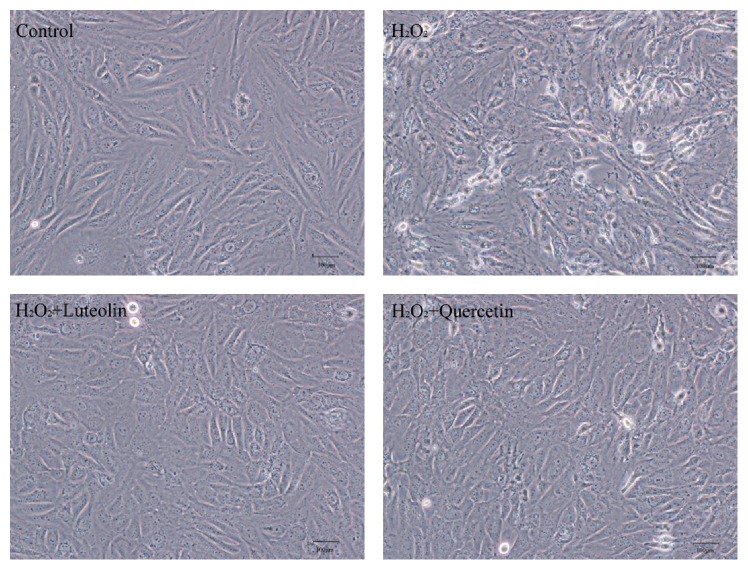
Morphologic changes of H9C2 cells induced by H_2_O_2_ with/without luteolin. H9C2 cells were preincubated with/without luteolin (10 *μ*M)/quercetin (10 *μ*M) for 6 h and then treated with 100 *μ*M H_2_O_2_ for 1 h. Cell morphology was examined under an inversion microscope.

To further evaluate the protective effect of luteolin against H_2_O_2_-induced cellular injury, cell morphology was examined under an inversion microscope. Normal cells in control group showed long fusiform shapes with clear structure (Figure 4). Treating cells with H_2_O_2_ (100 *μ*M) for 1 h resulted in cell shrinkage, alterations of cell shape, and wider intercellular gap. Pretreatment with luteolin and quercetin protected cells from the morphological changes induced by H_2_O_2_. These results demonstrated that luteolin pretreatment had significant protective effects against H_2_O_2_-induced cytotoxicity in H9C2 cells.

### 3.4. Luteolin Attenuated Intracellular ROS Generation

**Figure 5:**
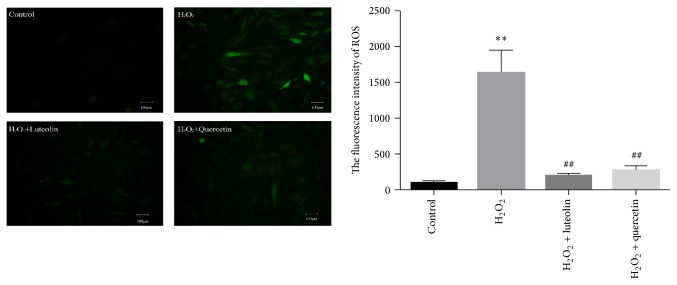
Intracellular ROS of H9C2 cells induced by H_2_O_2_ with/without luteolin. H9C2 cells were preincubated with/without luteolin (10 *μ*M)/quercetin (10 *μ*M) for 6 h and then treated with 100 *μ*M H_2_O_2_ for 1 h. Then cells were detected with DCFH-DA assay. Fluorescent intensity was examined under a fluorescence microscope. Image J was used to analyze the data. Data were expressed as mean ± SD. ^*∗∗*^ *P* < 0.01 versus control; ^##^ *P* < 0.01 versus model.

ROS generated by H_2_O_2_ could promote cellular damage. To determine whether pretreatment with luteolin could alleviate H_2_O_2_-induced early-stage oxidative stress, the treated cells were detected with DCFH-DA assay. Fluorescent intensity was examined under a fluorescence microscope. As shown in Figure 5, intracellular ROS significantly increased in H_2_O_2_-treated H9C2 cells compared with that in the control group. After pretreatment with luteolin (10 *μ*M)/quercetin (10 *μ*M), intracellular ROS was significantly reduced compared with that in H_2_O_2_-treated cells.

### 3.5. Luteolin Protected H9C2 Cells from H_2_O_2_-Induced Apoptosis

**Figure 6:**
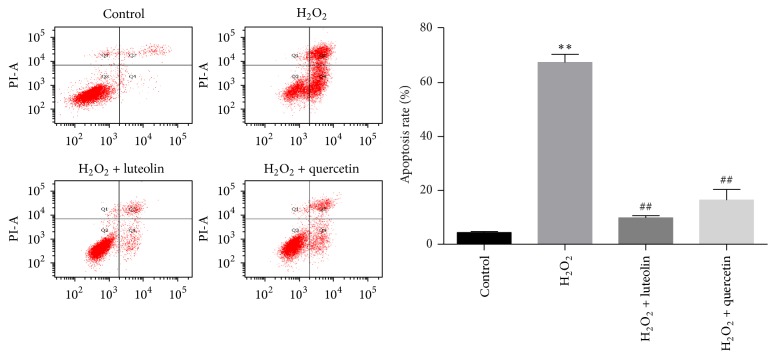
Luteolin protected H9C2 cells from H_2_O_2_-induced apoptosis. H9C2 cells were preincubated with/without luteolin (10 *μ*M)/quercetin (10 *μ*M) for 6 h and then treated with 100 *μ*M H_2_O_2_ for 1 h. Cells were harvested and detected with a flow cytometer. Data were expressed as mean ± SD. ^*∗∗*^ *P* < 0.01 versus control; ^##^ *P* < 0.01 versus model.

Apoptosis is considered to be one of the pathogenesis of H_2_O_2_-induced injury. To analyze the effects of luteolin on apoptosis in H9C2 cells, Annexin V-PI method was used to detect the apoptosis rate of H9C2 cells. As shown in Figure 6, the number of apoptotic cells significantly increased in H_2_O_2_ group compared to control group (67.47 ± 3.05% versus 4.33 ± 0.4%, *P* < 0.01). Pretreatment with luteolin/quercetin (10 *μ*M) for 6 h significantly reduced the percentage of apoptotic cells to 9.8 ± 0.85%/16.4 ± 3.96% (^##^ *P* < 0.01), suggesting that luteolin could protect H9C2 cells from apoptosis induced by H_2_O_2_.

### 3.6. Effects of Luteolin on the mRNA Expression of Mdm2

**Figure 7:**
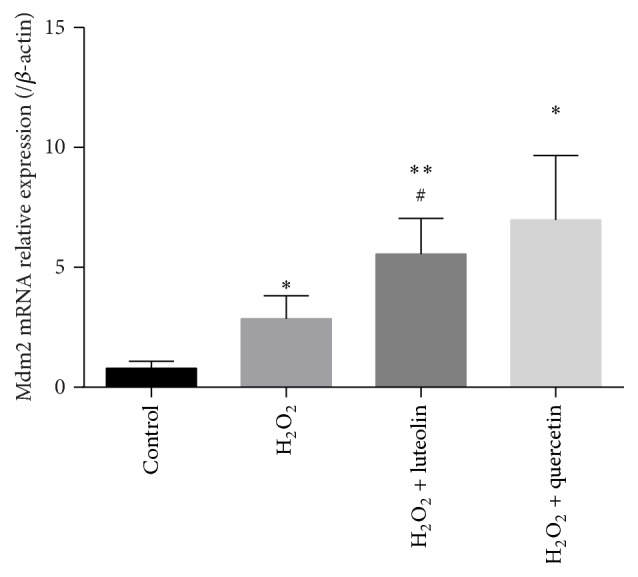
Luteolin increased the mRNA expression of Mdm2. H9C2 cells were preincubated with/without luteolin (10 *μ*M)/quercetin (10 *μ*M) for 6 h and then treated with 100 *μ*M H_2_O_2_ for 1 h. Cells were extracted, cDNA was synthesized, and the Mdm2 gene mRNA amount was assessed by quantitative real-time PCR and normalized to the *β*-actin. Data were expressed as mean ± SD. ^*∗*^ *P* < 0.05 and ^*∗∗*^ *P* < 0.01 versus control; ^#^ *P* < 0.01 versus model.

Mdm2, an E3 ubiquitin ligase, can be induced by increased cellular level of p53 and in turn negatively regulate p53 stability. When cells are exposed to H_2_O_2_, apoptosis is induced and expression of p53 is enhanced [20], as well as Mdm2. As shown in Figure 7, mRNA level of Mdm2 in H_2_O_2_ group increased slightly compared to control group (*P* < 0.05). Pretreatment with luteolin could remarkably elevate expression of Mdm2 (*P* < 0.05).

### 3.7. Effects of Luteolin on the Expression of Apoptosis-Related Proteins

**Figure 8:**
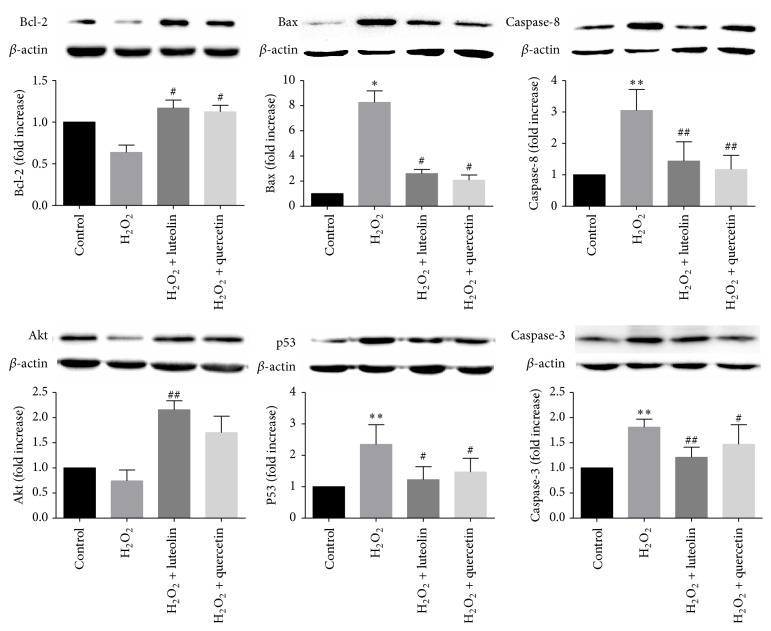
Luteolin modulated apoptosis-related proteins. H9C2 cells were preincubated with/without luteolin (10 *μ*M)/quercetin (10 *μ*M) for 6 h and then treated with 100 *μ*M H_2_O_2_ for 1 h. The expression of *β*-actin was measured as an internal control. Data were expressed as mean ± SD. ^*∗*^ *P* < 0.05 and ^*∗∗*^ *P* < 0.01 versus control; ^#^ *P* < 0.05 and ^##^ *P* < 0.01 versus model.

We further investigated the mechanism of luteolin in protecting cells from H_2_O_2_-induced apoptosis. Expressions of key molecules in apoptotic signaling pathway were measured by western blot. Bax, caspase-8, cleaved-caspase-3, and p53 are proapoptotic proteins. Akt and Bcl-2 have antiapoptotic effects. As shown Figure 8, in H_2_O_2_ group, expressions of Bcl-2 and Akt decreased, whereas the expressions of Bax, caspase-8, cleaved-caspase-3, and p53 increased compared to control group, indicating that apoptotic signaling pathway was activated by H_2_O_2_. Pretreatment with luteolin reversed the expressions of these proteins back toward normal levels. The results showed that protection of luteolin against H_2_O_2_-induced cell apoptosis may be mediated through Akt-p53 apoptotic signaling pathway.

## 4. Discussion

Luteolin is a plant flavonoid that exists in a variety of plants and has been shown to have antitumor, antioxidant, antiapoptotic, and anti-inflammatory effects [13–15]. Luteolin has protective effects against cardiovascular diseases, but the mechanism remains unclear. In this study, we investigated the effects of luteolin on apoptosis induced by H_2_O_2_ in H9C2 cardiomyocytes and explored the antiapoptotic mechanism. Our study showed that one of the antiapoptotic mechanisms of luteolin was mediated through Akt-p53/Mdm2 signaling pathway.

Studies have shown that apoptosis of cardiac myocytes is an essential process in the progression of heart failure [5]. A study using transgenic mice that expressed a conditionally active caspase demonstrated that low level of apoptosis is sufficient to induce lethal cardiomyopathy and inhibition of cell death can largely prevent the development of HF [21]. Antiapoptotic treatment is likely to become an important form of HF therapy [22].

Previous study showed that H_2_O_2_ could induce apoptosis in H9C2 cells [23]. In this study, we also demonstrated that H_2_O_2_ has proapoptotic effect on H9C2 cells. Pretreatment with luteolin could increase cell viability and reduce intracellular ROS level and apoptosis rate suggesting that luteolin possesses antioxidant and antiapoptotic properties in cardiomyocytes [24, 25]. Quercetin was applied as positive control drug in this study and it showed similar protective effect as that of luteolin. The cell survival rate in quercetin group was significantly higher than that in H_2_O_2_-treated group, indicating that quercetin also has antiapoptotic effects. We further explored the mechanisms by which luteolin exerts antiapoptotic effects.

Extrinsic and intrinsic signaling pathways can both lead to apoptosis. In the extrinsic pathway, caspase-8 activates caspase-3 after it is activated by intracellular and extracellular stimuli [26]. In the intrinsic pathway, permeability of mitochondrial membrane increases and proapoptotic factors, such as cytochrome C, are released to cytoplasm, activating signaling cascades and leading to activation of caspase-3 [27, 28]. The Bcl-2 family proteins, consisting of death antagonists (Bcl-2 and Bcl-xL) and death agonists (Bax and Bak), are also pivotal regulatory components of the cellular apoptosis process [29]. Their primary functions are to protect or disrupt the integrity of mitochondrial membrane and control the release of (pro)apoptotic proteins [5]. Our study showed that H_2_O_2_ induced apoptosis by downregulating Bcl-2 and upregulating expressions of proapoptotic proteins, such as Bax, caspase-8, and cleaved-caspase-3. In cells that were pretreated with luteolin/quercetin, expression of Bcl-2 was upregulated and expressions of Bax, caspase-8, and cleaved-caspase-3 were downregulated compared to apoptotic model cells, indicating that luteolin protects cells from apoptosis by modulating anti-(pro)apoptotic proteins.

Apoptosis is an orderly regulated process and p53 is one of the most important modulators in apoptotic signaling pathways [6, 30, 31]. Apoptosis induced by p53 can be divided into two types: the transcription-dependent apoptosis and transcription-independent apoptosis [32]. P53 interacts with transcription coactivator CBP/P300, induces apoptotic mRNA expression in the nucleus, and leads to the transcription-dependent apoptosis. In addition, p53 can bind to antiapoptotic Bcl-2 family proteins, such as Bcl-XL and Bcl-2, and release the proapoptotic effectors Bak/Bax from the complex, thereby inducing transcription-independent apoptosis. Regulating the level of p53 may be an effective target in the prevention of HF.

The activity and stability of p53 are regulated by a variety of enzymes and Mdm2 is one of the most important regulators. Mdm2 inhibits the transactivation ability of p53 by interacting with p53 transactivation domain and decreases the stability of p53 by promoting p53 ubiquitination [32]. Overexpression of Mdm2 exerts antiapoptotic effect and alleviates cardiomyocytes hypertrophy by inhibiting activity of p53 [10]. Ubiquitination activity of Mdm2 can be enhanced when Mdm2 is phosphorylated by Akt [33]. Therefore, activation of Akt could inhibit apoptosis by promoting degradation of p53. In the meantime, elevated cellular level of p53 can induce expression of Mdm2, which in turn suppresses activation of p53. The level of p53 is thus strictly controlled by this feedback loop between p53 and Mdm2 [9, 10]. Studies showed that, in tumor cells, luteolin exerts anticancer effects and promotes cell death by upregulating p53 expression [15, 34]. Luteolin can also exert antiapoptotic properties in cardiovascular diseases. However, the regulatory effect of luteolin on p53/Mdm2 in cardiomyocytes has rarely been reported. Our study showed that p53/Mdm2 pathway was altered in H_2_O_2_-treated cells. Expression of p53 was upregulated and expression of Akt was downregulated by stimulus of H_2_O_2_, leading to cellular apoptosis. Pretreatment with luteolin rescued cells from apoptosis by increasing expressions of Mdm2 and Akt and suppressing expression of p53. Pretreatment with quercetin only decreased the expression of p53 and had no significant effects on the expressions of Mdm2 and Akt. The results indicated that the antiapoptotic effect of luteolin is mediated by regulating Akt/Mdm2/p53 signaling pathway in H9C2 cells.

Treatment of H9C2 cells with H_2_O_2_ could induce cellular apoptosis. ROS level was increased and Akt-p53/Mdm2 apoptotic signaling pathway was activated in H_2_O_2_-treated cells. Pretreatment with luteolin could protect H9C2 cells from H_2_O_2_-induced apoptosis. The antiapoptotic effect of luteolin was mediated by upregulating expression of Bcl-2 and downregulating apoptotic proteins, including Bax, caspase-8, and cleaved-caspase-3. Furthermore, luteolin could suppress expression of p53 by activating Mdm2 and Akt. Our results illustrate that Akt-p53/Mdm2 signaling pathway is a potential target of luteolin in the prevention of HF.
